# Challenges using AI systems in the professions: the need for Hierarchical Team-based Intelligent Systems (HTIS)

**DOI:** 10.1080/12460125.2026.2666611

**Published:** 2026-05-13

**Authors:** Vicky Arnold, Phil Collier, Stewart A. Leech, Steve G. Sutton

**Affiliations:** a NHH Norwegian School of Economics, University of Central Florida, Orlando, FL, USA; b Department of Accounting, University of Melbourne, Parkville, VIC, Australia

**Keywords:** Artificial intelligence, Hierarchical Team Intelligent System, explainable AI, deskilling, intelligent systems, explanation facilities

## Abstract

Artificial intelligence (AI) is a significant challenge for the professions (e.g. auditing, consulting, engineering and law) as techniques hold great promise for improving effectiveness and efficiency of engagement performance, but techniques also pose challenges based on current technologies. Our research focuses on using AI embedded Hierarchical Team Intelligent Systems (HTIS). Engagements are completed traditionally with teams including novices (staff/seniors) and experienced/experts (partners/managers), but contemporary AI systems are designed for individual use. We propose a strategy for implementing AI in teams addressing several critical issues: novices with limited knowledge needing to input quality data, evidence that novices become less skilled when using AI, concerns over unexplainable AI and how to allow partners/managers to understand how decisions are formulated, and complexities over documenting AI inputs and decisions. Design recommendations provide potential solutions for these concerns, and behavioural testing with professionals supports the robustness of the various solution components in our design.

## Introduction

Rapid advances in artificial intelligence (AI) have reignited substantial interest among professional firms (e.g. auditing, consulting, engineering, law, etc.) and researchers on the human/AI relationship and AI techniques that can be leveraged to improve efficiency and effectiveness (Munoko et al., 2020; Sutton et al., 2018, 2023). Prior research has focused on an individual professional’s relationship with AI, a professional’s willingness to rely on AI and AI effects over time on users (e.g. Commerford et al., 2022; Munoko et al., 2020; Sutton et al., 2018, 2023). For professional firms, another level of complexity must be addressed – how can AI systems be effectively implemented in a hierarchical engagement team environment? In practice, these systems invariably become integrated into engagement workflow systems that direct engagement teams’ workflow (Dowling & Leech, 2007), facilitate work completed by staff/seniors to be easily reviewed by partners/managers (Dowling & Leech, 2014) and maintain a document trail and structured processes yielding a consistent methodology that meets regulators’ expectations for consistent process quality (Dowling & Leech, 2014; Zhang, Thomas, et al., 2022). AI systems add challenges in effectively applying appropriate techniques to the engagement and add complexity in effectively integrating these techniques into engagement workflow processes (Zhang, Cho, et al., 2022).

The purpose of our paper is to document an instantiation of an AI-based system, INSOLVE, that forms the basis of a Hierarchical Team Intelligent Systems (HTIS)^1^ and to address several issues that must be overcome to effectively integrate AI processes. We apply a design science methodology aligning with contemporary research perspectives on leveraging the benefits of both *design science* and *behavioural science* methods to systematically identify underlying problems, develop design solutions and test those solutions with appropriate professionals (Sutton et al., 2021). Literature increasingly recognises that major design science efforts are not accomplished through a single study but require a programme of research to systematically address design theory issues (Brocke et al., 2020; Gregor & Hevner, 2013). Further, when artefacts^2^ relate to systems interacting with humans, design theory validation should include behavioural testing of the effects on users (Hevner et al., 2004; Sutton et al., 2021). This study’s AI-system design is, accordingly, the product of a programme of research conducted with the insolvency profession in Australia over more than two decades with input and participation by partners/managers from multiple insolvency firms, as well as participation from many staff/seniors in testing.

Early in the research programme, multiple problems were identified forming the motivation for the subsequent research programme. First, for efficiency reasons, staff/seniors must complete most evidence gathering for AI-system input, but research highlights the negative impacts that arise when professionals work with systems that are more advanced than themselves. Less experienced professionals risk being unable to accurately capture and input the required data (Arnold & Sutton, 1998; Arnold, Collier, et al., 2004; Sutton et al., 2023). Using advanced technologies without fully understanding what the system is doing can also impede staff/seniors’ knowledge development (Dowling et al., 2008; Sutton et al., 2023).

Second, with risks during evidence gathering and entry, partners’/managers’ ability to review entries are key, but AI systems rarely document data entered. Partners/managers not only need to review entries, they also need to understand how AI makes decisions and how evidence is used in those decisions – the explainable AI challenge (Sutton et al., 2018, 2023; Zhang, Thomas, et al., 2022). Ideally, AI systems should be able to explain to staff/seniors how to properly enter information while also being able to explain the logic and use of information to partners/managers. Throughout, AI systems must collect and retain information entered to preserve documentation trails required by professional standards (Zhang, Thomas, et al., 2022). Adding complexity, team members are often dispersed; systems must support distributed teams.

We developed an AI system, INSOLVE, that operates over the Internet with a host server that (1) configures aspects of the AI-system interactions, (2) captures the entry of engagement evidence in a database, (3) provides comprehensive explanations adhering to theoretical guidelines on explanation types (definitions, rules, justification, strategic) and delivery mode (feedforward for entry, feedback for understanding outcomes), (4) dynamically generates tailored explanations using current engagement evidence and (5) enables instant replay of all prior entries by team members into AI systems. In short, INSOLVE addresses each issue identified during the Problem Identification phase. At each stage of development, staff/seniors and managers/partners participate in training sessions where INSOLVE is used to solve cases, including extensive reconstruction of actual engagements to validate and refine design theory components developed during the research. In the following pages, we elaborate on design choices, technological solutions, behavioural testing and validation and final prototyping of INSOLVE into a deployable-configurable HTIS.

## Research method

**Figure 1.**
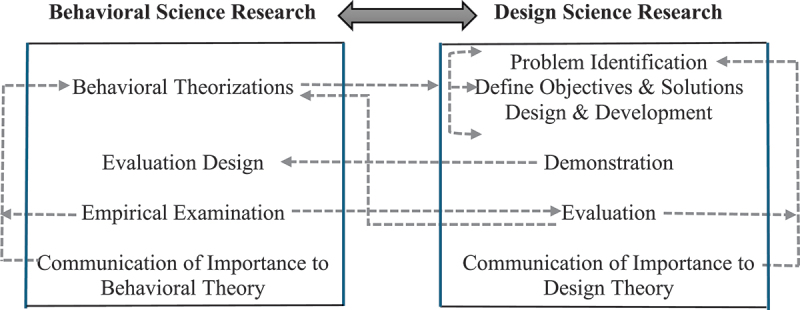
Leveraging the synergies between behavioural science and design science research.

The design science research method has evolved substantially over the past few decades with emphasis on enhancing the rigour of approaches employed and structuring the process (Hevner et al., 2004; Sutton et al., 2021). Sutton et al. (2021) specifically emphasise the need to include scientific examination with humans to further verify artefacts (Figure 1).

**Figure 2.**
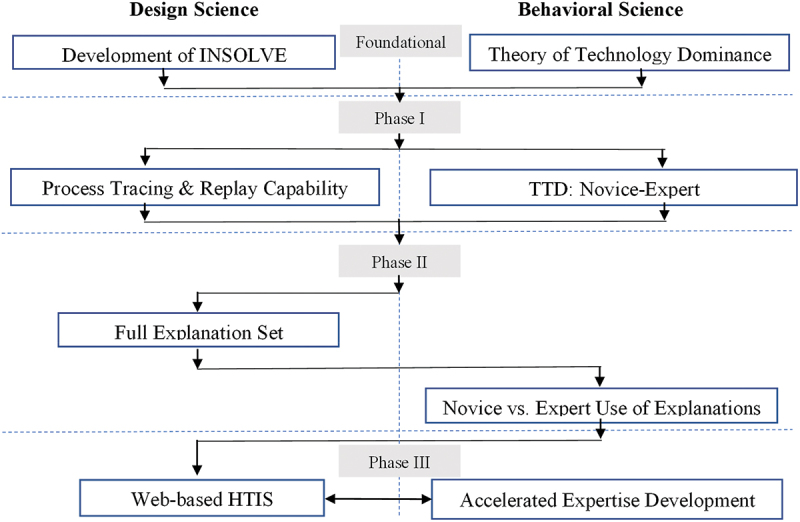
The INSOLVE project.

The interplay between design science and behavioural science should be extensive. Behavioural theory enables identifying problems and deriving design solutions. Demonstration of the instantiation is a standalone contribution to design theory, but evaluation is important in assessing the validity and robustness of the solution. Recent research emphasises that when the solution is an artefact intended to facilitate human–computer interaction, a critical component of evaluation is the application of behavioural analysis to understand how humans interact with systems and the resulting benefits/detriments (Kelton & Murthy, 2023; Sutton et al., 2021). Ultimately, design science contributions are assessed through the resulting artefact’s relevance and novelty, along with the contribution to design theory (Gregor & Hevner, 2013; Sutton et al., 2021). The INSOLVE project sought to apply both design science and behavioural science techniques to develop an artefact that would improve AI use in the professions (see Figure 2).

## Problem identification, solution and verification

Contemporary IS literature increasingly argues that research and practice need to recognise technology as the centerpoint and the human as an agent of the system – the system controls decision processes and the human is an artefact used by the technology (Demetis & Lee, 2018; Schuetz & Venkatesh, 2020). However, as technology advances, the professions such as insolvency cannot adopt such a model – the human maintains responsibility for decisions made (Sutton et al., 2023; Zhang, Thomas, et al. 2022). This necessitates that insolvency firms (and all professional firms) focus on adaptation of emerging AI technologies to balance techniques with the need for humans to interact and maintain control. A basic requirement of the design effort is that the AI system must be collaborative, allowing human and computer to trade-off control of the decision-making process but giving the human the ultimate decision (Arnold & Sutton, 1998; Sutton et al., 2023). This collaborative systems approach has also been referred to as the ‘electronic colleague’ (Arnold & Sutton, 1998). The INSOLVE project’s focus has always been on this collaborative systems approach.

From a research standpoint, identifying a domain where the professional firm has a narrow focus was critical to being able to develop a working prototype and to be able to identify specific professionals with focused expertise in the area. We chose the insolvency environment in Australia, where like most countries evolving under British law, accounting professionals are tasked with administrating companies in known financial distress with weak going-concern status. The insolvency professional has control over decisions to liquidate, sell off portions of the business or to reconstruct and hand back to owners/management. Decisions are complex and risky and involve human judgement, but the outcome possibilities are limited, making insolvency a useful environment for academic research. If an engagement partner/manager/director continues an insolvent business, they can be responsible for additional losses. Thus, only expert professionals can really succeed in the profession (Arnold, Collier, et al., 2004).

### Problem identification

Problem Identification is the initial stage in the research process where problems are defined, problem relevancy is assessed and deficiencies in current solutions are assessed. The research programme began with the development of a working AI prototype that mimics the cognitive processes and decision-making of insolvency experts (based on 23 partners’/managers’ decision-making patterns) (Collier et al., 1999; Leech et al., 1998, 1999). This is a traditional use of AI where systems mimic human judgement based on environmental cues to assess risks, make predictions and suggest action (Munoko et al., 2020).

The original INSOLVE was tested experimentally with 37 partners/managers and 43 staff/seniors using INSOLVE to gain familiarity with the system and complete an insolvency engagement case.^3^ The original experiment focused on testing for hypothesised effects from the Theory of Technology Dominance (Arnold & Sutton, 1998), predicting increased decision bias and error from novices (staff/seniors) using AI, while experts (partners/managers) were expected to benefit from collaboration with the system, reducing bias and errors in decisions. We used traditional behavioural experimentation to explore decision biases focusing on the bias towards the most recent information received (order-recency bias). Theorised effects held, highlighting risks of novices overreacting to new evidence and over-adjusting estimates entered into the AI system, but demonstrating the ‘electronic colleague’ benefit of pairing AI with an expert by reducing the overreaction to recent information (Arnold, Collier, et al., 2004).

Post-experiment, discussion groups were conducted to identify what worked well, what were the limitations of the AI system and perspectives on collaborative interaction. Novices made it clear the system did not represent how they viewed decisions being made as they worked primarily with financial statement analyses and that was not a significant part of the system. From the experts (partners/managers), there was a translation of the novice perspective – novices focused on lower-level financial information, while experts focused on higher-level qualitative assessments of the business and management. The experts’ discussion led to a question of whether we (1)‘*could design INSOLVE so novices learned during usage and improved their insolvency knowledge?*’ On a second dimension, one expert noted he found the system highly useful and he would like to have it as an assistant in his work, *but he would never take the time to enter all of the evidence into INSOLVE himself*. Time was too crucial. The experts coalesced around this idea in agreement and the solution was viewed as, (2) ‘*could the researchers set-up INSOLVE so novices could enter the information and partners/managers could simply review the information and decision outcome?*’ A third dimension related to the level of benefit for experts as partners/managers coalesced around the desire for more explanation of INSOLVE’s decision—(3) ‘*the decision output with explanation was useful, but we would like to be able to dig deeper into understanding how INSOLVE derived the decision and what evidence supported that decision?*’ The problem identification process, combining experimental results with debriefing discussions, indicated that AI supporting hierarchical teams was needed and several challenges must be addressed to overcome the identified problems.

### Defining solutions→Developing artefact→Evaluating→Repeat

#### Making AI explainable

The primary challenges are interrelated in terms of the research streams on *explanation facilities* and *explainable AI*. Explanation facilities allow users to explore an AI system’s reasoning when anomalies are encountered, users want to learn or users need to understand a concept to participate properly in problem solving (Gregor & Benbasat, 1999). Explainable AI is the ability of an intelligent system to explain its AI-driven decisions (Zhang, Cho, et al., 2022).

As an intermediary step in the development of the current INSOLVE, we focused on modifying the original single user INSOLVE to form a testbed for the development of an effective explanation function. The research on explanation facilities provides a framework for explanation type and presentation mode to facilitate explainable AI (Gregor & Benbasat, 1999). Explanation types include *Definition, Rule-trace, Justification* and *Strategic*. Presentation mode relates to *feedforward* versus *feedback* (Arnold, Clark, et al., 2004). Feedforward assists AI users when entering information to understand what information is being requested, how it will be used, and the decision path the AI is seeking. These are static explanations as they are generic statements regardless of the evidence entered. Feedback explanations allow the user to interpret outcomes from AI and can include static explanations for definitions of terms in outcome descriptions but are mostly dynamic where evidence processed by the AI becomes part of the explanations to clarify how decisions were derived, justify decision-making processes and interpret conclusions (e.g. those needed for *explainable AI*). However, prior studies used very limited subsets of these explanation types without working AI systems – thus ignoring feedforward explanations that help the user when entering data and reacting to system queries. We approached the study with the belief that fully working systems and comprehensive explanation facilities were necessary to truly study users’ behaviour.

INSOLVE included a full set of explanations using all four types and both feedforward and feedback modes (a total of 1,119 explanations were linked into the system). To develop justification and strategic explanations, residual knowledge from the knowledge acquisition process with the 23 experts was integrated with knowledge engineered into the AI to increase the robustness (Arnold, Clark, et al., 2004).

The extended INSOLVE was evaluated through human interaction. An experiment working on an insolvency engagement case was conducted with 27 partners/managers and 39 staff/seniors. We captured each entry by the user including explanation access with participants having explanations available on a voluntary use basis, and the data were compared with the original data to observe differences in patterns of use and decision outcomes.

Results showed staff/seniors used more feedforward explanations (understanding evidence to be entered into the AI system and how that information will be used) and lower-level knowledge (definitions and rule-traces). Partners/managers used more feedback explanations (how and why the decision outcome was derived by the AI) and high-level knowledge (justification and strategic). Partners/managers were also more likely to agree with INSOLVE recommendations after viewing feedback explanations (Arnold et al., 2006). While all effects were statistically significant, the number of explanations voluntarily viewed was low and overall use was not particularly common. While the array of explanations appeared to meet the needs of different skill levels of users, the desired effects may be muted by low use.

#### Constructing an HTIS: making AI work effectively in hierarchical teams

The results of the intermediary steps were positive and insightful, but further adjustments seemed warranted. Adjustments were not major, and we integrated changes in developing the AI system underpinning INSOLVE as a necessary foundation for a deployable-configurational HTIS. One adjustment was automatically providing explanations versus leaving them as only voluntary. A key issue was that novices appeared better served with different explanations than experts, yet both should have access to whatever explanations they desired.

**Figure 3.**
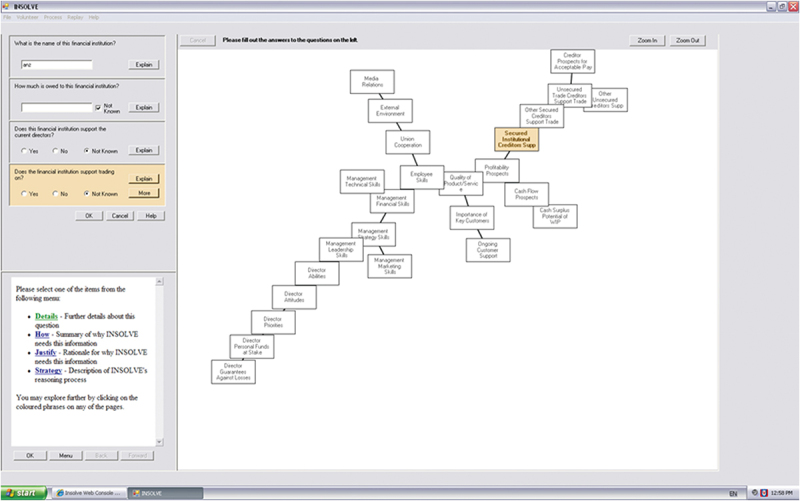
HTIS screenshot with cognitive map interface.

To accomplish this change and move towards a hierarchical-team architecture, we built an Internet-based deployable system using a central control system to identify team members who are allowed access to the client engagement instance, but also allowed user profiles to be set by experience/expertise. These profiles (1) facilitated determination of explanations provided by INSOLVE, focusing on lower-level knowledge explanations for novices and higher-level knowledge explanations for more senior team members. (2) We added the capability to alter screen layouts based on individual users. The screen layout consists of three panels (see Figure 3). The upper left panel provides data input controls and in turn the recommendations generated by INSOLVE. A second panel below the data entry/recommendation panel provides explanations to the user based on their level of expertise. Explanations can be automatic or voluntary (the latter shown in Figure 3). The right panel of the screen is a segment that can be altered in INSOLVE through a simple mapping process. For novice users, the map as shown in Figure 3 is designed to visualise the cognitive relationships of the various concepts used by the AI system as determined through a knowledge mapping process completed by a set of experts (partners/managers).^4^

Research has shown that such a mapping interface can enhance novices’ expertise level while using the system, despite users being unaware of the interface’s role (Rose et al., 2007). Such maps can have negative effects on users already possessing expert-level understanding of the relationships (Rose et al., 2007). Accordingly, for more experienced users, we used a generic alphabetical listing of concepts rather than the expert cognitive map.^5^

Profiles can also be set to limit access to certain AI functionality. For instance, a staff member working on the support from financial institutions for the client’s continuance of operations may only have access to that part of the evidence provision, whereas another staff member may have permissions only for addressing support from suppliers.^6^ This allows team coordinators to provision access in a way that promotes the best quality input to an AI system.

Regardless, evidence gathered in the system must be tracked for review at the manager/partner level. INSOLVE’s central control is designed to capture each entry made during an engagement using the system to create a logged history of all entries, including any subsequent changes to previously entered information. These changes are important in terms of viewing how evidence evolves with the business’ situation and whether decisions about the future of the business in financial distress need to change.

The control system also provides a ’resume session’ module (Arnold et al., 2001). Data entered as evidence is pulled from a server database storing the logged data entries and uses entries to chronologically repopulate INSOLVE and restore it to its prior state. The resume module has multiple speeds, including step-by-step clicking through each evidence entry sequentially to allow partners/managers to review evidence gathered and entered into the system and understand the data driving the outcome recommendation. (Alternatively, the current state of the INSOLVE processing system can be instantaneously restored, without the visualisation process.) The underlying database also provides an audit trail allowing the work to be later reviewed for quality control reasons or regulatory requirements.

The new HTIS version of INSOLVE was designed to improve novice performance levels, avoid deskilling effects on novices that have been theorised in the literature and observed in some cases, but also to allow novices to funnel better quality inputs into the INSOLVE reasoning model for use by the experts making the critical decisions. Therefore, the partners/managers making key decisions needed to see usefulness in the system, but we also needed to understand whether the intended improvements in novice performance materialised.

#### Examining the human–HTIS interaction

INSOLVE HTIS was similarly evaluated through scientific examination with the human. However, testing in a true hierarchical interaction was not feasible and we divided the testing into two solo phases. In the first phase, a dozen partners/managers participated in a one-hour session where they completed a tutorial case using INSOLVE and one additional self-complete case. The tutorial assisted participants in exploring all components of INSOLVE to understand capabilities. The feedback was very positive on INSOLVE with specific reference to usefulness of the explanations for understanding decision processes, particularly when engagements had different outcomes than businesses they normally engaged. The partner/manager participants also committed most of their 1–3-year experience-level staff for participation in 3-day sessions to evaluate novice interactions with the HTIS.

We obtained 67 staff-level participants that completed the full training (Arnold et al., 2023). The training consisted of tutorials on using the case simulation software and INSOLVE, followed by six reconstructed engagements using INSOLVE to assist in decision-making. In debriefing, participants were overwhelmingly enthusiastic about how much they learned about the overall engagement process and the benefits of case-based training (we first surveyed on traditional measures of usability and satisfaction followed by an open discussion and debriefing session). However, our research interest was not just in how the AI system affected engagement completion but in how it reshaped the user’s cognitive processes—*could we avoid deskilling the novices and hopefully actually skill them during productive work use?*

The sessions were structured such that we divided participants into four groups, with two groups receiving an instance of INSOLVE configured to provide automatic explanations and two groups receiving an instance of INSOLVE configured to provide the cognitive map interface (one group with neither, one group with both). We focused on the development of cognitive schema and understanding relationships among concepts driving decision-making.

Our design attributes showed significant improvements. Participants receiving automatic explanations or those with the cognitive map interface significantly improved their cognitive schema (based on how they mapped the concept relationships before and after the training), with participants moving reasonably close to the expert’s consensus mapping (i.e. experts’ schema). When provided both, the improvement was even greater – but when neither was provided, a small, significant decrease in cognitive structure was observed. This decrease is consistent with deskilling concerns associated with novices (staff/seniors) using AI systems (Arnold & Sutton, 1998; Arnold, Collier, et al., 2004; Sutton et al., 2023). These findings support the robustness of our design strategies in developing an effective concept for the design of HTIS.

## Summary

In this study, the focus is on developing a design concept and prototype for an AI-based system that can be used in a hierarchical team environment where expert professionals are responsible for final decision-making, but remotely located novice professionals are responsible for aggregating and entering the data used by the AI system. There are multiple challenges in designing such a system. First, the system must be configured to the user given the different needs of experts and novices in successfully using AI. Second, AI has been shown to have risks of increasing decision bias and deskilling novices. Third, the system must log all entries for review by experts as in a professional firm setting as they face legal and regulatory concerns over decisions. Fourth, the system must be designed as a distributed AI-based system to support a geographically dispersed team.

An AI-based system prototype developed through a series of design science studies and related behavioural science examination addresses these challenges. INSOLVE HTIS contains a central control system that manages a distributed AI system allowing each user instance to be tailored to the user’s expertise level. For novices, this includes the provision of a knowledge map interface and automatic explanation provision – an interface we show to mitigate deskilling concerns and assist the novice in effective data provision. All data entries are captured and retained in a central database to allow review access by experts making decisions and keeping a long-term record of how the AI was used.

There are additional facets of the system that would benefit from further research. First, all data entries are retained in the system database, but making this information accessible in a format best for the experts would further refine the concept. Second, the prototype utilises more traditional explainable AI and research into maintaining the benefit of our concept while also adapting emerging AI methods would further advance design flexibility.

## Data Availability

This study uses Design Science Methodology based on a programme of prior research. As a Design Science study, there is no data to make available. Please see prior publications.

## Notes

1. We define a Hierarchical Team Intelligent System as an AI-based system that is designed to be used with teams of professionals where some team members have supervisory responsibilities over other team members’ work, generally with an experience/expertise differential among the team members.

2. An artefact is defined as any designed object in which a research contribution is embedded in the design, including determining the desired functionality and its architecture. Artefacts can be represented by constructs, models, methods and instantiations (Gregor & Hevner, 2013; Hevner et al., 2004).

3. We wanted to differentiate between experts (partners/managers) and novices (senior/staff) to understand the differential needs. In professional firms, partners and managers oversee engagements, make key decisions and have legal responsibility. Senior and staff are less experienced staff that do the evidence gathering and more routine tasks such as financial estimates and financial analyses.

4. The display shows a coloured tile activated to mirror the question being asked in the AI processing box to help visualise the connection to the factors presented in the knowledge map interface.

5. A cognitive map lays out the relationship between key concepts (data) with the distance between concepts relating to the closeness of the relationship between concepts. These maps tightly relate to the schema a decision maker uses in organising information. If novices can be moved closer to expert’s schema, expertise development can be accelerated (Arnold et al., 2023).

6. This was not implemented in INSOLVE, rather for simplicity in the prototype we allowed access to the full system by users during validation and locked active use to one user in an engagement at a time.
